# Artificial intelligence methods enhance the discovery of RNA interactions

**DOI:** 10.3389/fmolb.2022.1000205

**Published:** 2022-10-07

**Authors:** G Pepe, R Appierdo, C Carrino, F Ballesio, M Helmer-Citterich, PF Gherardini

**Affiliations:** ^1^ Department of Biology, University of Rome “Tor Vergata”, Rome, Italy; ^2^ PhD Program in Cellular and Molecular Biology, Department of Biology, University of Rome “Tor Vergata”, Rome, Italy

**Keywords:** RNA, RNA interaction predictors, natural language processing, deep learning, machine learning, embedding, RNA sequence, RNA secondary structure

## Abstract

Understanding how RNAs interact with proteins, RNAs, or other molecules remains a challenge of main interest in biology, given the importance of these complexes in both normal and pathological cellular processes. Since experimental datasets are starting to be available for hundreds of functional interactions between RNAs and other biomolecules, several machine learning and deep learning algorithms have been proposed for predicting RNA-RNA or RNA-protein interactions. However, most of these approaches were evaluated on a single dataset, making performance comparisons difficult. With this review, we aim to summarize recent computational methods, developed in this broad research area, highlighting feature encoding and machine learning strategies adopted. Given the magnitude of the effect that dataset size and quality have on performance, we explored the characteristics of these datasets. Additionally, we discuss multiple approaches to generate datasets of negative examples for training. Finally, we describe the best-performing methods to predict interactions between proteins and specific classes of RNA molecules, such as circular RNAs (circRNAs) and long non-coding RNAs (lncRNAs), and methods to predict RNA-RNA or RNA-RBP interactions independently of the RNA type.

## Introduction

The involvement of RNAs in a wide range of biological processes, such as transcription, translation, neurogenesis, and the biogenesis and function of non-coding RNAs (ncRNAs) has been discussed in multiple studies (Newman et al., 2015; Turner and Díaz-Muñoz, 2018; Peng et al., 2022). Basic cellular physiology is critically dependant on RNA-Protein interactions (RPIs), as exemplified by their role in RNA splicing, transcription efficiency, stabilization and termination (Kelaini et al., 2021), in triggering RNA release from the transcription complex (Van Assche et al., 2015), and in regulating RNA degradation (Gilbertson et al., 2018). RNAs interact with RNA-Binding Proteins (RBPs) through sequence and structural motifs (Dominguez et al., 2018). Adinolfi et al. (Adinolfi et al., 2019) identified several RNA binding motifs by analyzing PAR-CLIP, eCLIP and HITS-CLIP experiments. Starting from these motifs, Guarracino et al. developed a web server for the identification of enriched structure or sequence motifs in a pool of RNAs which returns putative interacting RBPs (Guarracino et al., 2021). Altered functionality of RBPs and subsequent disruption of RNA-RBPs regulatory networks are commonly observed in human genetic diseases, neurodegenerative diseases and multiple cancer types (Pereira et al., 2017; Gebauer et al., 2021; Schieweck et al., 2021). Besides interacting with proteins, RNAs can also interact with each other, giving rise to complex regulatory networks that control cellular physiology in health and disease (e.g. mRNA regulation exerted by miRNA) (Chen et al., 2019; Pepe et al., 2022b; Wang et al., 2022). Moreover, RNAs influence each others’ expression level by competing for a limited pool of microRNAs (miRNAs) (Seitz, 2009; Poliseno et al., 2010), as postulated by the “competitive endogenous RNA” (ceRNA) theory (Salmena et al., 2011). The interaction between viral DNA or RNA genomes and host miRNAs is involved in immune system evasion and viral replication (Qiao et al., 2019). Accordingly, the role of exogenous DNA or RNA in viral infection has been extensively studied, highlighting how viral genomes can act as “sponges” for specific host miRNAs. This mechanism has been described for Hepatitis C Virus (Luna et al., 2015) and Epstein-Barr Virus (Riley et al., 2012) and it has also been suggested for SARS-CoV-2 (Pepe et al., 2022a).

Given the importance that RNA interactions play in fundamental cellular processes, cancer, and other diseases, several methods for studying the physical interactions between RNA and proteins have been developed (Ferrè et al., 2015). These *in vitro* or *in vivo* methods can be classified into two main categories: i) RNA-centric methods used to study proteins associated with a specific RNA; ii) protein-centric methods used to identify RNAs interacting with a specific protein (Ramanathan et al., 2019). Despite the large number of RNA interactions identified thanks to these methods, experimental validation is still expensive and time-consuming and computational approaches remain an active area of research.

In this review, we aim to elucidate recent advances in RNA interaction predictions, focusing on state-of-the-art methods currently used for the prediction of RNA-RNA or RNA-RBP interactions. The development of these methods is critically dependent on the quality and characteristics of datasets of known interactions. Accordingly, we will also review publicly available sources of RNA interaction data.

## Overview of databases

A crucial element in the development of RNA-protein interaction prediction models is the retrieval of datasets containing known interacting pairs to be used for ML models’ training. The present section will therefore survey two fundamental aspects in this respect. Firstly, we describe the main features of the most widely employed datasets for RNA-protein interaction prediction. Indeed, during the last decade, various datasets have been constructed and released to pursue this task. Such datasets, typically, rely on information maintained in databases or obtained through literature-mining operations and they involve interactions supported by experimental evidence. Subsequently in this section, a second crucial aspect is pointed out. Since machine learning methods for binary classification need to be trained on datasets containing a balanced number of samples from both classes to be predicted, in the case of RPI prediction this translates into disposing of datasets containing RNA-protein pairs that are known to interact (which will henceforth be referred to as “positive dataset”) as well as non-interacting RNA-protein pairs (which will henceforth be referred to as “negative dataset”). We reported an overview of the major methods employed for the construction of RPI negative sets as well as a summary table reporting assumptions and outlines of such strategies.

### Publicly available datasets of RNA interactions

Datasets currently considered as benchmarks for training, cross-validating or testing RPI prediction models include RPI369 and RPI2241 (Muppirala et al., 2011), RPI488 (Pan et al., 2016), and RPI1807 (Suresh et al., 2015). These are structure-based datasets which incorporate interaction pairs obtained from RNA-protein complexes whose structures have been deposited in the PDB (Velankar et al., 2021). Another commonly used dataset is NPInter2.0 (Yuan et al., 2014), which contains interactions derived from literature-mining and other databases.

The RPI2241 and RPI369 datasets were obtained from PRIDB (Lewis et al., 2011), a database of protein-RNA interfaces derived from PDB complexes (Burley et al., 2021). A total of 943 complexes from PRIDB (9,689 protein chains and 2,074 RNA chains) were initially selected. A final dataset consisting of 2241 experimentally validated RNA-protein interacting pairs (952 protein chains and 443 RNA chains) was derived, by redundancy reduction (discarding similar interaction on the basis of sequence identity) and sequence length filtering. When the RPI2241 dataset was constructed, a sizable fraction of all the RNA-protein complexes in the PDB corresponded to ribosomal structures, leading to a strong bias towards ribosomal RPIs. Accordingly, a second dataset, RPI369, was generated from RPI2241 by removing all RPIs that contained ribosomal proteins or ribosomal RNAs. Moreover, to generate a balanced dataset of non-interacting RNA-protein pairs, the RNAs and proteins from the original 943 complexes were randomly paired and pairs similar to known interactions were further discarded.

The RPI488 dataset is a structure-based dataset, derived from PDB complexes and specifically incorporating lncRNA-protein interactions. In order to generate the dataset, 18 ncRNA-protein complexes were downloaded from the PDB and 726 lncRNA-protein pairs were collected from them. In order to derive both a positive and a negative dataset, a distance cutoff of 5Å was used. Also, redundant sequences (sequence identity greater than 90% for both protein and lncRNA sequences) were excluded by using CD-HIT (Fu et al., 2012). Following redundancy reduction, the final RPI488 dataset contains 488 protein-lncRNA pairs (243 interacting pairs and 245 non-interacting ones).

The RPI1807 dataset was derived by integrating the Nucleic Acid Database (NDB) (Coimbatore Narayanan et al., 2014) and the PRIDB. A total of 1560 RPI complexes available in NDB were selected and, for 1336 of them, atomic interactions were extracted from PRIDB, thus obtaining 13,163 protein and 2715 RNA chains. The procedure for constructing the dataset included sequence length filtering and redundancy removal according to sequence similarity. In order to obtain both positive and negative sets, the selected non-redundant pairs were further analyzed for atomic interactions with a distance threshold (3.40 Å). This threshold was used to distinguish strongly interacting protein-RNA pairs (positive set) from weakly interacting protein-RNA pairs (negative set). The final RPI1807 dataset consists of 1807 positive pairs and 1436 negative pairs.

**FIGURE 1:**
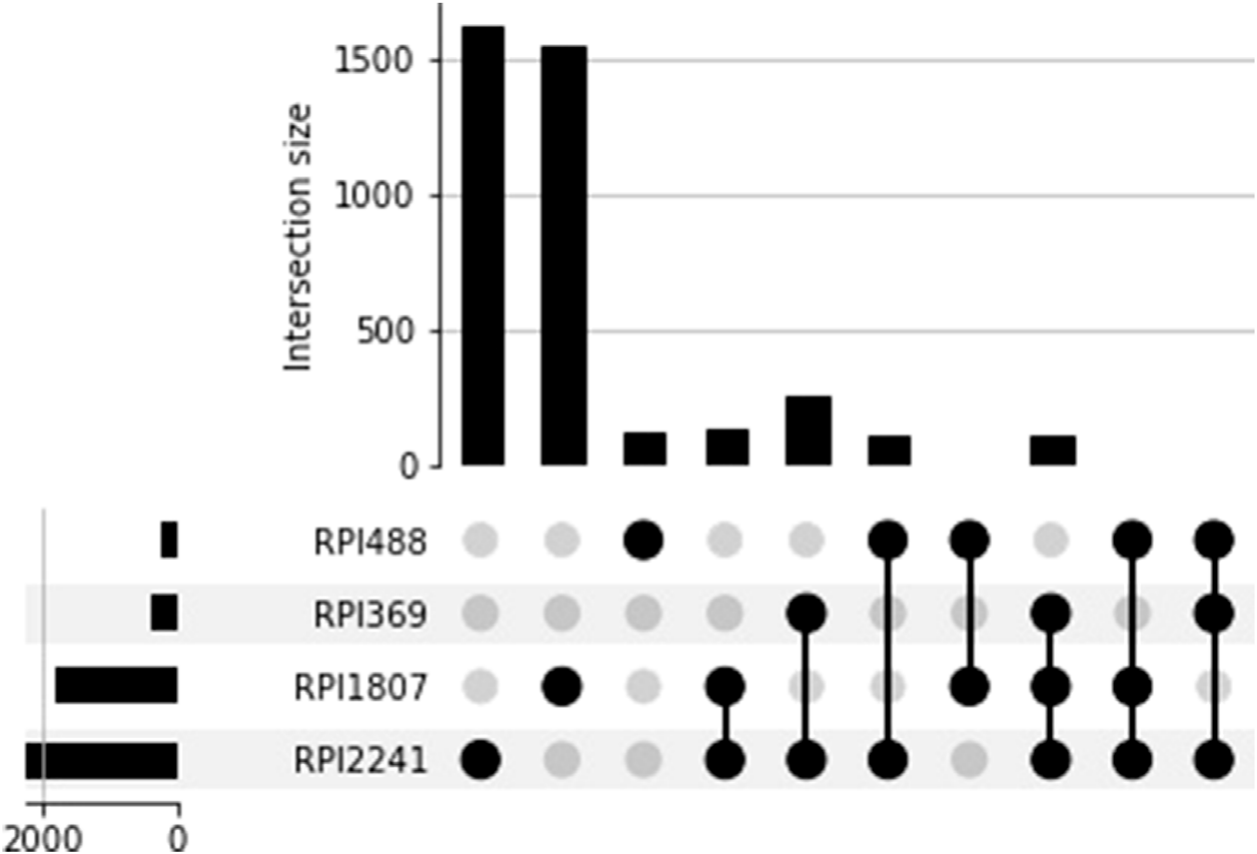
Overlap between the four RPI datasets generated from PDB RNA-protein complexes.

The overlap between RPI datasets is reported in Figure 1. This overlap could be greater than that obtained by simply intersecting the RNA-protein pairs since a redundancy reduction was applied to each one of the RPI datasets. In each of the RPI datasets, RNA-protein pairs were clustered and only one pair was chosen as representative; this could influence the overlap between the four datasets.

NPInter2.0 is a database that integrates experimentally-validated functional interactions between ncRNAs and other biomolecules (RNAs, proteins and DNAs), collected both from literature mining and from multiple databases. Although newer releases of the database exist (up to NPInter v4.0), NPInter v2.0 is the most widely used dataset for the development of prediction models. The dataset contains a total of 201,107 ncRNA interactions from 18 organisms, excluding interactions involving tRNAs and rRNAs.

Interactions were derived from manual annotation of articles published between 2008 and 2013 and include both experimentally-validated interactions as well as binding sites identified by genome-wide techniques (Yuan et al., 2014). The authors also integrated data from other resources, mainly the LncRNADisease database (Chen et al., 2013), and finally performed a redundancy reduction procedure within the dataset.

Several datasets have been derived from NPInter2.0 by selecting subsets of interactions with characteristics of interest. More specifically, the most widely used non-structure-based dataset for the development and testing of RPI prediction models is a subset of this database (namely NPInter10412) first assembled by Suresh et al, (2015), and subsequently used in numerous other works (Li et al., 2021; Wang et al., 2021; Zhao et al., 2021). NPInter10412 contains 10,412 ncRNA-protein interactions, distributed among the different species as illustrated in Table 1.

**TABLE 1:** Number of RNA-protein interactions by species in the NPInter10412 dataset.

Species	# of RNA-protein interactions
*H. sapiens*	6975
*M. musculus*	2198
*D. melanogaster*	91
*C. elegans*	36
*S. cerevisiae*	905
*S. cerevisiae S288c*	5
*E. coli*	202

A fourth release of NPInter was published in 2019 that increases the amount of high-throughput interactomes available data. NPInter4.0 (Teng et al., 2019) includes 600,000 new ncRNA interactions, particularly ncRNA–DNA interactions obtained *via* the ChIRP-seq technique, as well as interactions involving circular RNAs. Additionally, disease associations were added to the database.

Lastly, RNAInter4.0 is a recent resource that integrates experimentally validated and computationally predicted RNA interactions from literature-mining and databases (Kang et al., 2022). It provides information about different types of interactions in different *taxa*. Tables 2 and 3 summarize RNAInter’s content.

**TABLE 2:** Number of interactions of each type in the RNAInter database.

Interaction type	# of interactions
RNA-RNA	9,483,936
RNA-Protein	37,060,698
DNA-RNA	138,552
RNA-Histone modification	1,060,684
RNA-Compound	10,889

**TABLE 3:** Number of interactions in the 8 taxa in the RNAInter database. An overview of the main publicly available datasets for RNA-protein interaction prediction is given in Table 4.

Taxon	# of interactions
Actiniaria	872
Arthropoda	538,643
Bacteria	72,132
Fungi	622,927
Nematoda	883,131
Vertebrata	45,584,924
Viridiplantia	58,875
Virus	712,704

**TABLE 4:** Publicly available datasets for RNA-protein interaction prediction.

Dataset	# Of positive interactions	# Of negative interactions	Description	Negative set strategy	References
RPI2241	2241	2241	Structure-based dataset, containing RNA-protein interactions enriched in ribosomal RPIs	Random Pairing	Muppirala et al. (2011)
RPI369	369	369	Structure-based dataset, obtained from RPI2241 after removal of interactions derived from ribosomal complexes	Random Pairing	Muppirala et al. (2011)
RPI488	243	245	Structure-based dataset, comprising interactions between proteins and different classes of RNAs	Least atom distance	Pan et al. (2016)
RPI1807	1807	1436	Structure-based dataset, comprising interactions between proteins and different classes of RNAs	Least atom distance	Suresh et al. (2015)
NPInter10412	10,412	-	Non structure-based dataset, comprising RNA-protein interactions integrated from literature mining and other databases	-	Yuan et al. (2014);
					Suresh et al. (2015)

Ultimately, despite remarkable advances in experimental techniques, the development of large and reliable RPI datasets is still the main bottleneck for training ML models. Hence, we would also like to stress the importance of redundancy control within data, since its presence may cause a leakage of information between training and test set during model training, resulting in untruthful prediction performance.

### Strategies for the construction of a negative dataset

The lack of reliable datasets of non-interacting RNA-protein pairs is a major concern in the development of computational methods for RPI prediction. Indeed, it is not trivial to conclusively state that a given protein does not interact with a given RNA molecule (absence of evidence does not constitute evidence of absence). Indeed, various papers have demonstrated the critical effect of negative dataset composition on the performance of Machine Learning and Deep Learning models (Muppirala et al., 2011; Pan et al., 2016; Peng et al., 2019). Additionally, having balanced positive and negative sets is crucial to avoid overfitting on one class.

The most often used (Muppirala et al., 2011; Pan et al., 2016; Yi et al., 2020) method to construct a dataset of non-interacting pairs is to randomly pair RNAs and proteins in the positive set, followed by discarding the thus obtained pairs that showed high sequence similarity to the interacting ones, while retaining the others.

An interesting, albeit not widely used, technique to construct negative samples is the FIRE (FInding Reliable nEgative samples) method (Cheng et al., 2017). The core idea of this method relies on the following observation: given an experimentally-validated interaction between protein p1 and RNA r, and given another protein p2, the more similar p2 is to p1, the higher the likelihood that r interacts with p2. Thus, for each positive RPI (p1, r) the p2 protein that is most dissimilar to p1 is selected; if (p2, r) is not an experimentally-validated RPI, then it is selected as a negative RPI. The innovation introduced in this work lies in the way the similarity between each pair of proteins was computed, by taking into account functional annotations and protein domains information in addition to sequence similarity.

An additional approach that circumvents the requirement to create a negative dataset is PU learning, a binary classification method that can be applied when only positive (P) and unlabeled (U) data are available. For example, PRIPU trains a biased SVM on only positive and unlabelled examples (Cheng et al., 2015, 2017).

Some of the most often employed strategies for the construction of a negative dataset are listed in Table 5.

**TABLE 5:** Strategies for the construction of a negative dataset for RNA-protein interaction prediction.

Strategy	Assumption	Description
Random pairing	The likelihood of interaction occurring between randomly paired RNAs and proteins is low	By using known interacting pairs as starting point, the same number of non-interacting pairs are generated by randomly pairing RNAs and proteins from the positive set, followed by discarding pairs that are similar to interactions already present in the positive set
FIRE method	Given a known RNA-protein interacting pair (p1, r), and given a second protein p2, the smaller the sequence similarity between p1 and p2, the lower the likelihood that r interacts with p2	For each positive RNA-protein interaction (p1, r) the p2 protein that is most dissimilar to p1 is selected, similarity between each pair of proteins was computed by taking into account functional annotations and protein domain information in addition to sequence similarity
Subcellular localization method	RNAs and proteins that are not in the same subcellular compartment do not interact with each other	This method requires subcellular localization data
Least atom distance criterion	Only applicable to interactions derived from known-structure complexes	Given a multimolecular RNA-protein complex, for each pairwise combination of its constituent RNA and protein molecules, if there is at least one atom of the RNA located closer than a threshold to at least one protein atom, the pair is considered to be interacting otherwise it is included in the negative dataset

## Computational methods for RNA-protein interaction discovery

If on the one hand, the choice of the right training dataset is critical, on the other hand the choice of the right algorithm for RNA-RBP interaction prediction is also important, considering that some predictors were developed for a specific class of RNAs, such as lncRNA or circRNA. We will therefore review the latest methods for RNA-RBP interaction prediction. Some methods predict interactions between proteins and a specific class of RNA molecules, such as circRNAs (Yang et al., 2021; Niu et al., 2022) and lncRNAs (Ge et al., 2016; Zhao et al., 2018; Xie et al., 2019; Zhou et al., 2021). Others were developed to predict RNA-RBP interactions independently from the RNA type (Akbaripour-Elahabad et al., 2016; Yi et al., 2018; Zhan et al., 2018; Wang et al., 2019, 2021; Zhang et al., 2020).

### LPI-deepGBDT: An artificial intelligence algorithm for the prediction of long non-coding RNA-protein interactions

Long non-coding RNAs (lncRNAs) are a class of RNA molecules that have attracted strong interest in recent years due to their abundance and their role in many physiological and pathological processes (Kornienko et al., 2016). Since many of the functions performed by lncRNAs require their interaction with proteins (LPIs), and most of lncRNAs are of unknown function, identifying new LPIs is a very important task. Most of the methods developed for this task are based on hand-crafted features, which is a process that requires time, domain knowledge and is based on strong assumptions. We describe the LPI-deepGBDT algorithm (Table 6), which uses a feed-forward deep architecture based on gradient boosting decision trees (Zhou et al., 2021). In this work three human and two plant LPI datasets, derived from the NPInter database, were used as training for the classifier. These datasets were processed using several filters, similar to previous works (Li et al., 2015; Zheng et al., 2017; Zheng et al., 2017; Zhang et al., 2018; Bai et al., 2019). Multiple features of lncRNAs and proteins were calculated from their sequences using Pyfeat (Muhammod et al., 2019) and BioProt (Márquez and Castro Amaya, 2019). The dimensionality of the feature space was then reduced using PCA, and protein and RNA features were concatenated to obtain a matrix of features representing the interaction pairs. This matrix was used as input to the classifier, which consisted of a multi-layered deep framework based on a gradient boosting model. The authors compared their model with five state-of-the-art LPI prediction methods, namely LPI-BLS, LPI-CatBoost, PLIPCOM, LPI-SKF and LPI-HNM (Yang et al., 2016; Deng et al., 2018; Fan and Zhang, 2019; Wekesa et al., 2020; Zhou et al., 2020), using six measurements: precision, recall, accuracy, F1-score, AUC and AUPR, and obtaining better average performances. Furthermore, the LPI-deepGBDT algorithm was successfully applied to the identification of potential protein partners for a specific lncRNA and, given a specific protein, to infer its potential interacting lncRNAs. The authors highlight that one of the main drivers of performance improvement for this method is the integration of biological features.

**TABLE 6:** Description of the train/test datasets, feature encoding and machine learning strategy for each of the described methods.

Method	Interacting molecules	Train/test dataset	Feature encoding	Machine learning strategy	References
LPI-deepGBDT	lncRNA-RBP	Derived from NPInter	Sequence features extracted using Pyfeat (Muhammod et al., 2019) and BioProt (Márquez and Castro Amaya, 2019)	Gradient boosting decision trees	Zhou et al. (2021)
LncPNet	lncRNA-RBP	Derived from NPInter v2.0	Heterogeneous network embedding of lncRNAs and proteins similarity networks and of the known lncRNA-protein interaction network	Support-vector machine	Zhao et al. (2021)
CRBPDL	circRNA-RBP	CLIP-seq experiments	k-nucleotide frequency (KNF), Doc2vec, electron-ion interaction pseudopotential (EIIP), chemical characteristics of nucleotides (CCN) and accumulated nucleotide frequency (ANF)	Deep multi-scale residual network (ResNet) and bidirectional gated recurrent unit with a self-attention mechanism (BiGRUs)	Niu et al. (2022)
EDLMFC	ncRNA-RBP	RPI1807 NPInter v2.0 RPI488	k-mer frequencies of the sequence and structure representations	Ensemble deep learning framework including convolutional neural networks (CNN) and bi-directional long short-term memory net-work (BLSTM)	Wang et al. (2021)
preMLI	miRNA-mRNA	Plants lncRNA-miRNA interaction dataset constructed using RNAHybrid 2.1.2	word2vec based sequence embedding	CNN and bidirectional gated recurrent unit (Bi-GRU)	Yu et al. (2022)
PrismNet	RNA-RBP	CLIP-seq experiments	One-hot-encoded sequence vectors and icSHAPE structure scores	Convolutional layers, squeeze-and-excitation networks (SE) and residual blocks	Sun et al. (2021)
PRNA	RNA-RBP	RsiteDB	Number of atoms, electrostatic charge, potential hydrogen bonds, hydrophobicity and relative accessible surface area were used as sequence features. Secondary structure of amino acid residues, conservation score (PSI-BLAST), side-chain environment were used as structure features. A sliding window was used to encode amino acid residues and create feature vectors	Random Forest	Liu et al. (2010)

### LncPNet: A human long non-coding RNA-protein interactions predictor

Most models are developed to predict lncRNA-protein interactions irrespective of the species, which can result in the introduction of noise and negatively affect performance.

To address this and other limitations, Zhao et al, (2021) introduced a new predictor model called LncPNet (Table 6). This method is designed to exclusively predict human lncRNA-protein interactions. Moreover, protein and lncRNA features are automatically generated using a network embedding. For this study, human lncRNA-protein interactions were selected from NPInter v2.0 resulting in 7523 experimentally validated pairs, including 3052 lncRNAs and 212 proteins. LncRNAs and proteins lacking sequence information were removed, thus obtaining a dataset of 4578 interactions between 2009 lncRNAs and 78 proteins. The negative dataset was built using the subcellular localization method (see Table 5). This method is based on a heterogeneous network of lncRNA-protein which is constructed using: i) lncRNA-lncRNA and protein-protein similarity; ii) known lncRNA-protein association. The similarity between lncRNAs and proteins is both calculated by Jaccard similarity and BLAST similarity. Subsequently the metapath2vec (Dong et al., 2017) method is used for network embedding and dimensionality reduction. LncRNA-protein interactions are represented as vectors of dimensionality 1 x 256 and those vectors are used to train a Support Vector Machine in order to predict whether an lncRNA interacts with a protein. Comparison with other state-of-the-art methods shows that LncPNet achieves better performances in terms of accuracy, F1-score and MCC.

### CRBPDL: A deep learning approach for the prediction of circular RNA-RBP interactions

Circular RNAs or circRNAs are non-coding RNA molecules which can bind RBPs and are involved in multiple regulatory processes (Zang et al., 2020). CRBPDL (Table 6) (Niu et al., 2022) is a recently developed method that uses a deep learning approach (also used in other studies, e.g. Pan and Shen, 2018; Zhang et al., 2019; Yang et al., 2021) to predict interactions between circRNAs and proteins. The main improvement of CRBPDL is in the feature encoding step, which is critical for prediction performance. CRBPDL uses five different coding schemes (k-nucleotide frequency, Doc2vec, electron-ion interaction pseudopotential, nucleotide chemical properties, and cumulative nucleotide frequency) for the construction of a feature matrix. The method then uses a deep neural network architecture in order to extract local and global context information and subsequently train the model with a self-attention mechanism checking the robustness of the method. The deep neural network architecture is composed by a ResNet (a deep multi-scale residual network) and a BiGRUs (bidirectional gated recurrent unit) with the final integration of AdaBoost algorithm in order to improve the prediction performances. The authors trained and benchmarked CRBPDL using a circRNAs-RBPs interaction dataset derived from the CircInteractome database (Dudekula et al., 2016), consisting of interactions from 37 CLIP-seq experiments, consistently obtaining better performances when compared with existing methods. CRBPDL encodes different types of information about the sequence of circRNA: the dinucleotide and trinucleotide composition frequency (KNF), the free electron energy (EEIP), and also chemical informations about the nucleotides that compose circRNA sequences. For long-term context dependencies Doc2vec, used as encoding scheme, demonstrated to give a great contribution to the feature representation. CRBPDL was also tested on 31 datasets of linear RNA-RBP interactions, obtaining an average AUC of 0.91, which is significantly higher than the AUCs of other methods (ICIRCRBP-DHN (Yang et al., 2021), CRIP (Zhang et al., 2019), iDeepS (Pan et al., 2018), and CIRCSLNN (Ju et al., 2019)). CRBPDL is available on Github (https://github.com/nmt315320/CRBPDL).

### EDLMFC: An ensemble deep learning framework for the prediction of non-coding RNA-RBP interactions

In this section, we discuss a class of ncRNA-RBP interaction predictors not designed for a specific RNA type. A recent computational method developed in this field, called EDLMFC, uses an Ensemble Deep Learning framework with Multi-scale Features Combination (Table 6) (Wang et al., 2021). EDLMFC was trained on ncRNA-RBP interaction pairs derived from the RPI1807, NPInter v2.0, and RPI488 datasets and uses different types of features as input such as the primary sequence and the secondary and tertiary structure of ncRNAs and proteins. Using a greater number of features was shown to increase prediction performance compared with single features. This method combines two different techniques: i) a convolutional neural network (CNN); ii) a bi-directional long short-term memory network (BLSTM). The first one is a deep learning-based method which is used to extract high-level information from the features and the second one is a recurrent neural network method which learns long-range dependencies between features, mainly on sequential data. Finally, a three-layer, fully connected, layer is able to predict ncRNA-protein interactions. In a five-fold cross-validation experiment, EDLMFC obtained better performance than RPITER (Peng et al., 2019), IPMiner (Pan et al., 2016), and CFRP (Dai et al., 2019). Moreover, independent tests demonstrated that EDLMFC can be effectively used to predict potential ncRNA-protein interactions in different organisms.

### PRNA: Binding site features enable improvement RNA-protein interaction prediction

For the prediction of RNA-RBP interactions, several methods have been developed in order to find the potential binding sites in RNA or in RBP sequences. One of them is from Liu et al, (2010) (Table 6). In this work the authors highlighted the importance of both sequence and structure features in RNA-binding proteins, that simultaneously contribute towards the recognition of a specific RNA sequence site. In order to determine in a more comprehensive way the interacting sites in protein sequences, the authors suggested a parameter to consider interaction propensity of an amino acid. This variable represents a measure of mutual dependence of a triplet of amino acids in proteins where the central amino acid binds a nucleotide on the RNA sequence. Then this feature is encoded in a vector of other hybrid features to describe exhaustively the amino acids in the protein sequence. The method was trained using a dataset of protein-RNA complexes obtained from RsiteDB and used to predict RNA binding residues in proteins given the previous set of features, using Random Forest (RF), that with a sliding window of 5 amino acids on the protein sequence predicts the possible site of a binding event. The result in terms of AUC is of 0.905 with a ACC of 81.4% indicating a good performance if compared to other methods (RNABindR (Terribilini et al., 2007), BindN (Wang and Brown, 2006), RNAProB (Cheng et al., 2008), PPRint (Kumar et al., 2008)). In this paper the idea emerges that by integrating the information carried by the neighborhood of an amino acid with other features of the protein sequence and structure analyzed, we can substantially improve the prediction of RNA-RBP interactions by finding the binding sites. A concept well developed also in a recent work of Niu et al. in which instead of focusing on the binding protein sequence, the RNA sequence is fundamental.

### PrismNet: A deep learning algorithm to predict RPIs that uses *in vivo* RNA structures

One of the most important factors determining the interaction between RNAs and proteins is the RNA secondary structure (Taliaferro et al., 2016). Therefore, leveraging this feature in prediction models can significantly increase their performance. Although there are different methods for the prediction of RNA secondary structure (Seetin and Mathews, 2012), computational methods based exclusively on the primary sequence do not take into account the dynamic nature of these structures. Indeed, RNA secondary structures are extremely dynamic and can change depending on various factors such as the interaction with chaperones and other RBPs. All these factors, ultimately, vary depending on the cellular conditions *in vivo* (Lewis et al., 2017). PrismNet is an RNA-protein prediction method that leverages experimental data on RNA secondary structures, being capable, in this way, to take into account their dynamism (Table 6). This method is based on secondary structure information obtained *via in vivo* click selective 2′-hydroxyl acylation and profiling experiments (icSHAPE) (Flynn et al., 2016) that were carried out in 7 cell types (i.e. K562, HepG2, HEK293, HEK 293T, HeLa, H9, and mES) in which RNA structures were profiled transcriptome-wide. This data was integrated with RBPs binding sites data from CLIP experiments in the same cell types. To construct the model input, the structure scores derived from the icSHAPE experiments were encoded as a one-dimensional vector and the sequence was represented as a four-dimensional one-hot-encoded vector. The deep learning model consists of a series of convolutional layers, while squeeze-and-excitation networks were used to recalibrate the convolutional channels and residual blocks to capture the joint sequence and structural determinants of RBP binding. The authors compared their model with other computational methods including RCK (Orenstein et al., 2016), GraphProt (Maticzka et al., 2014; Orenstein et al., 2016) and DeepBind (Alipanahi et al., 2015), using the binding sites obtained from the CLIP-seq datasets for each RBP, and obtaining better performance in terms of AUC and AUPRC. Furthermore, by training their model using different combinations of inputs, they observed that the model trained using both the sequence and the experimentally determined RNA secondary structures outperformed other models, demonstrating that experimental information on the RNA secondary structure *in vivo* is critical to the performance improvement.

## Computational methods for RNA-RNA interactions prediction

RNAs can also interact with other RNAs and several studies have shown these interactions to be crucially involved in the regulation of gene transcription, cell metabolism, and other key cellular functions (Deogharia and Gurha, 2021; Singh et al., 2022; Wang et al., 2022). Despite the fact that a large number of RNA-RNA interactions have been experimentally validated, many more have yet to be identified. Therefore, several computational methods have been developed for the prediction of RNA-RNA interaction, many of which are based on sequence complementarity (Kang et al., 2020, 2021; Yang et al., 2020). In the last 5 years, these methods have been gradually revolutionized by the introduction of deep learning approaches borrowed from the field of natural language processing. PreMLI is one of the latest methods in this field, it was published in early 2022 by Yu and collaborators (Table 6) (Yu et al., 2022), and, currently, it achieves better overall performance compared with other existing methods. This method was specifically built to predict miRNA-lncRNA interactions and relies exclusively on RNA sequence information. PreMLI was trained using a plant lncRNA-miRNA interaction dataset, constructed using RNAHybrid 2.1.2. The approach consists of three steps: i) in the pre-training phase the RNA sequences are used as input for rna2vec training in order to obtain a weight matrix that better describes the RNA sequence and can be used as the input to the next step; ii) deep feature mining approaches, based on Convolutional Neural Network, Bidirectional Gated Recurrent Unit, and attention layers are used to obtain additional potential features; iii) in the last step the two feature vectors are connected as input to the prediction layer. The authors demonstrate how the pre-training and the deep feature mining phases improve prediction performance and, furthermore, they show how this method performs better than already existing advanced RNA-RNA interaction predictors in terms of sensitivity, specificity, and AUC. Although the pre-training step improves the model performance, it also increases the computational time required for the entire prediction process. Moreover, this method is optimized for the prediction of miRNA-lncRNA interactions in plants. In order to extend its use to other types of RNA-RNA interactions or other organisms the model needs to be trained on an appropriate specific dataset and the hyperparameters need to be adjusted.

## Conclusion

In the last few years several studies have explored the RNA interactions landscape, given the crucial role that RNA-RBPs and RNA-RNA networks play in cell biology. Despite the advances made so far, novel experimental methods for the identification of binding sites (such as HITS-CLIP and PAR-CLIP) are still time-consuming and cost-intensive. That is why computational approaches represent a complementary strategy to guide experimental work. In this review, we provide an overview of the most recent prediction methods. We summarize recent advances in the algorithms developed to solve specific tasks, such as circRNA- or lncRNA-RBPs interaction predictions or, more generally RNA-RBPs interactions. Besides, we highlight how the development of a larger dataset of interactions is crucial to increase performance. Lastly, despite the fact that many methods rely only on sequence information, among the ones analyzed, those that obtain the best performances tend to include a variety of different biological features. Performance comparison of the described methods shows how the inclusion of structure information contributes to improving the accuracy and efficiency of the models. Only one of the described methods uses both RNAs and proteins structural information as input features for the predictive model because if, on the one hand, a large number of reliable protein structures is available, on the other hand, RNA structures are mainly obtained through computational prediction. RNA structure uncertainty could add noise to the model, resulting in untruthful prediction performances. The prediction of protein structure has reached satisfactory levels of performance thanks to the development of AlphaFold (Jumper et al., 2021). Conversely, RNA structure prediction still lags far behind. One of the main limitations is the paucity of known RNA structures that can be used for model training. To address this issue a new deep learning model called Atomic Rotationally Equivariant Scorer (ARES) has been developed (Townshend et al., 2021). ARES achieves good performances in the prediction of RNA structures, based on a training dataset of only 18 experimentally determined RNA structures. While this is a useful development, further work is needed in this area. Ultimately, as demonstrated by the methods described in this review, the availability of high-quality RNA structure predictions could greatly improve the inference of RNA-RBP and RNA-RNA interactions. Moreover, the advances in RNA secondary structure determination methods, that takes into account the information from biochemical assay like icSHAPE-seq (Flynn et al., 2016), could improve the confidence of such information as a feature for prediction models, likely leading to an improvement of their performance.
